# Navigating the Complexity: A Comprehensive Review of Hemophagocytic Lymphohistiocytosis Associated With Dengue Infection

**DOI:** 10.7759/cureus.61128

**Published:** 2024-05-26

**Authors:** Aman Gupta, Tushar Sontakke, Sunil Kumar, Sourya Acharya, Utkarsh Pradeep

**Affiliations:** 1 Medicine, Jawaharlal Nehru Medical College, Datta Meghe Institute of Higher Education and Research, Wardha, IND

**Keywords:** multidisciplinary management, treatment, diagnosis, hyperinflammation, dengue, hemophagocytic lymphohistiocytosis

## Abstract

Hemophagocytic lymphohistiocytosis (HLH) associated with dengue infection presents a unique challenge in clinical practice due to its rarity, rapid progression, and overlapping clinical features. This comprehensive review navigates the complexity of HLH-dengue syndrome by examining its pathophysiology, clinical manifestations, diagnostic criteria, and therapeutic strategies. HLH, characterized by uncontrolled immune activation and cytokine dysregulation, can occur as a secondary complication of dengue infection, leading to severe multiorgan dysfunction and high mortality if not promptly recognized and treated. The review underscores the significance of early diagnosis through vigilant clinical monitoring and appropriate diagnostic tests, such as bone marrow examinations and genetic studies. Collaboration between infectious disease specialists, hematologists, and critical care teams is essential for optimal management. Despite advancements in understanding HLH-dengue syndrome, further research is needed to elucidate its underlying mechanisms and explore novel treatment approaches. This review provides insights into the clinical implications of HLH-dengue syndrome and emphasizes the importance of a multidisciplinary approach to improve patient outcomes in this challenging clinical scenario.

## Introduction and background

Hemophagocytic lymphohistiocytosis (HLH) is a rare and life-threatening hyperinflammatory syndrome characterized by uncontrolled immune system activation [1]. It is marked by excessive cytokine release, tissue infiltration by activated lymphocytes and macrophages, and hemophagocytosis. HLH can be primary (genetic) or secondary (acquired), with secondary HLH being frequently triggered by infections, malignancies, or autoimmune disorders [2]. Dengue infection is a mosquito-borne viral illness caused by the dengue virus, transmitted primarily by Aedes mosquitoes [3]. Dengue presents a spectrum of clinical manifestations, ranging from mild febrile illness to severe forms such as dengue hemorrhagic fever (DHF) and dengue shock syndrome (DSS). The global burden of dengue has been increasing, with millions of cases reported annually in tropical and subtropical regions [4].

The association between HLH and dengue infection has gained attention due to its clinical significance and challenges in management. Dengue-induced HLH, although rare, can lead to rapid clinical deterioration and poor outcomes if not promptly recognized and treated. Understanding the interplay between dengue infection and HLH is crucial for optimizing patient care and outcomes [5]. This comprehensive review aims to provide insights into the complex relationship between hemophagocytic lymphohistiocytosis and dengue infection. It will delve into the pathophysiology, clinical manifestations, diagnostic challenges, and therapeutic strategies associated with HLH-dengue syndrome. Additionally, the review will highlight gaps in current knowledge and propose future research directions to enhance our understanding and management of this clinically important entity.

## Review

Hemophagocytic lymphohistiocytosis (HLH)

Definition and Classification

Hemophagocytic lymphohistiocytosis (HLH) is a rare and life-threatening condition characterized by an aberrant and hyperactive immune system response [1,6,7]. HLH can be categorized into two primary types: primary (familial) and secondary. Primary HLH arises from genetic mutations that impair the ability of white blood cells to combat infections. At the same time, secondary HLH develops due to another underlying illness, typically a widespread infection or immune disorder [8,9]. The clinical presentation of HLH includes marked inflammation and tissue damage affecting organs such as the liver, spleen, and bone marrow, with symptoms that may mimic severe infection or other medical conditions [8,9]. Diagnosis entails a series of blood tests, including a complete blood count, liver function assessments, and specialized tests to identify signs and causes of infection. Further diagnostic insights may be obtained through bone marrow or lymph node tissue samples, and genetic testing can reveal defective genes in primary HLH cases [8,9]. Specialized centers typically coordinate treatment for HLH with expertise in managing rare immune disorders. The primary objective of treatment is to dampen the excessive immune response, often achieved through administering corticosteroids and intravenous chemotherapy medications in a hospital setting [8,9]. In certain instances, corrective measures like hematopoietic stem cell transplant (HSCT) may be warranted to induce remission, particularly in cases of primary HLH [8,9].

Pathophysiology

HLH manifests as an aberrant and hyperactive immune response, culminating in profound inflammation and tissue injury [10-13]. A defining feature of this condition is the aggressive proliferation and activation of macrophages and histiocytes, which engulf other blood cells [12,13]. The classification of HLH primarily encompasses two categories: primary (familial) and secondary (acquired) forms [12,13]. Primary HLH arises from genetic mutations impairing the function of cytotoxic lymphocytes (natural killer cells and cytotoxic T cells), leading to persistent antigen stimulation and uncontrolled immune activation [10-13]. Conversely, secondary HLH emerges as a consequence of an underlying condition, such as widespread infection, autoimmune disorders, or malignancy [12,13]. In these instances, the primary disease triggers the immune system, precipitating the hyperinflammatory state characteristic of HLH [12,13]. Irrespective of the triggering factor, HLH culminates in a "cytokine storm," an excessive and uncontrolled release of inflammatory cytokines, including interferon-gamma, tumor necrosis factor-alpha, and interleukins [12,13]. This hypercytokinemia underpins the severe tissue damage and multi-organ failure observed in HLH [12,13].

Clinical Presentation

The clinical presentation of hemophagocytic lymphohistiocytosis (HLH) encompasses fever, hepatosplenomegaly, bicytopenia or pancytopenia, evidence of hemophagocytosis in the bone marrow and lymphoid tissues, liver dysfunction, and involvement of the central nervous system [14]. Patients afflicted with HLH commonly display a state of hyperinflammation, precipitating severe symptoms and potential complications [15]. Notably, in adults, the clinical manifestations of HLH may diverge from those observed in children, with lower incidences of hepatomegaly, splenomegaly, and jaundice but a heightened frequency of serous cavity effusion [14]. Laboratory assessments often reveal thrombocytopenia, elevated serum ferritin levels, and deviations in liver function tests [14]. Moreover, the time elapsed from the onset of symptoms to clinical diagnosis tends to be prolonged in adults compared to children, rendering the disease more challenging to identify in the adult population [14]. Consequently, recognizing hallmark clinical features such as fever, cytopenias, and organomegaly is imperative for facilitating timely diagnosis and implementing appropriate management strategies to enhance patient outcomes [6,14].

Dengue infection

Introduction to Dengue Virus

The dengue virus, belonging to the Flavivirus genus within the Flaviviridae family, is characterized by its positive-stranded RNA structure [16,17]. This virus comprises four distinct serotypes: DENV-1, DENV-2, DENV-3, and DENV-4, and exhibits antigenic differences [16]. Additionally, a fifth serotype, DENV-5, was recently identified in Malaysia in 2015 [16]. Transmission of the dengue virus primarily occurs through the bite of infected Aedes aegypti and Aedes albopictus mosquitoes, prevalent in tropical and subtropical regions where they breed in water-filled containers [16,18]. Dengue infection manifests across a spectrum of clinical presentations, ranging from asymptomatic cases to severe dengue hemorrhagic fever (DHF) and dengue shock syndrome (DSS) [16,18]. Typical symptoms of dengue include high fever, headache, body aches, nausea, and rash [16,18]. With an estimated 100-400 million infections annually, dengue poses a significant global public health challenge, particularly in tropical and subtropical regions [16,18,19]. The escalation in dengue incidence in recent years is attributed to population growth, urbanization, and climate variations [16,18]. Given the potentially life-threatening nature of severe dengue, prompt recognition and appropriate management in a hospital setting are imperative [18]. Specific antiviral treatments for dengue are currently unavailable, underscoring the importance of vector control measures and the development of effective vaccines for prevention [16,18].

Epidemiology and Transmission

Dengue fever, a viral infection transmitted by mosquitoes, notably Aedes aegypti and Aedes albopictus, is the fastest-spreading mosquito-borne viral disease globally [20,21]. The transmission dynamics of the dengue virus are influenced by a myriad of environmental factors, encompassing vector density, human mobility, and climatic conditions such as temperature, all of which impact the abundance and distribution of the mosquito vectors [21,22]. Humans serve as the primary amplifying host for the dengue virus, with female mosquitoes acquiring the virus from viraemic humans during blood meals. Subsequently, the virus infects the mosquito midgut, disseminates systemically, and transmits to other humans after an extrinsic incubation period [21]. Aedes aegypti, distinguished by its anthropophilic behavior, frequent biting tendencies, and proximity to human habitats, emerges as a highly efficient vector for dengue virus transmission. While laboratory studies have demonstrated vertical transmission of the virus, its significance for viral maintenance in natural settings remains uncertain [21]. The epidemiological landscape of dengue infections exhibits global variation, with regions like the Americas witnessing cyclical outbreaks recurring every three to five years. In this region, the circulation of all four dengue virus serotypes has engendered substantial reported cases and fatalities, notably exemplified by the significant outbreak in 2002 [3]. Within the European Economic Area (EU/EEA) region, dengue cases predominantly arise from travel to dengue-endemic countries across Africa and the Americas. Nonetheless, autochthonous cases have been documented, with Aedes albopictus serving as the principal competent vector for dengue virus transmission in mainland Europe [23].

Clinical Manifestations

**Figure 1:**
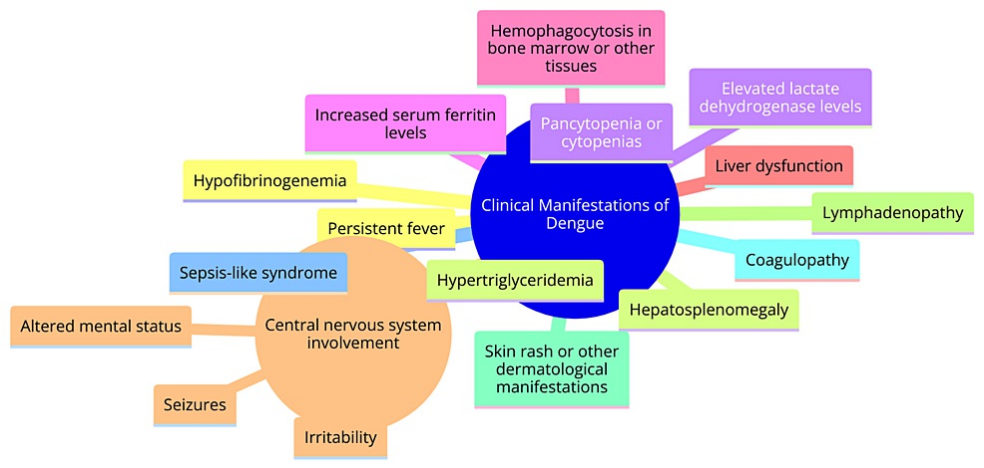
Clinical manifestations of dengue Image Credit: Dr Aman Gupta

The febrile phase of dengue infection is characterized by a sudden onset of high-grade fever, typically reaching around 40°C (104°F), which persists for two to seven days. Associated symptoms during this phase encompass facial flushing, skin erythema, myalgias, arthralgias, headache, sore throat, conjunctival injection, anorexia, nausea, and vomiting. In approximately 6% of cases, a saddleback or biphasic fever pattern may manifest, particularly in severe dengue [24]. The critical phase signifies the emergence of warning signs, including a rapid decline in platelet count, hematocrit elevation, and other concerning indicators. During this phase, patients may progress to complications such as shock, organ dysfunction, disseminated intravascular coagulation, or hemorrhage [25]. The subsequent recovery phase is distinguished by the gradual reabsorption of extravascular fluid over two to three days, often accompanied by bradycardia. Severe dengue represents a life-threatening complication characterized by warning signs such as abdominal pain, persistent vomiting, fluid accumulation, mucosal bleeding, lethargy, and hepatomegaly. These severe manifestations can precipitate profound bleeding, shock, and organ compromise. Timely recognition of warning signs is imperative to prevent the progression to severe disease [26]. The clinical spectrum of dengue infection spans from asymptomatic cases to severe, life-threatening complications such as dengue hemorrhagic fever and dengue shock syndrome. A comprehensive understanding of the distinct phases and prompt recognition of warning signs are pivotal for the timely and effective management of dengue patients [27]. Clinical manifestations of dengue are shown in Figure 1.

Diagnosis and Management

Dengue fever poses diagnostic challenges due to its clinical signs and symptoms, which can overlap with those of other vector-borne diseases such as malaria, chikungunya, and zika fever [28]. Consequently, laboratory confirmation of dengue virus infection is imperative, involving the detection of the virus, viral nucleic acid, antigens, or antibodies in the patient's blood [25,28]. Serological assays play a crucial role in diagnosis, particularly during the febrile phase, by detecting specific immunoglobulin M (IgM) or immunoglobulin G (IgG) antibodies to the dengue virus [28]. Additionally, testing for the NS1 antigen provides an early diagnosis in febrile patients, facilitating prompt identification of dengue infection [28]. As there is no specific treatment for dengue fever, management primarily revolves around supportive care, including rest, adequate fluid intake, and monitoring for signs of dehydration [25,28]. Patients with dengue fever are advised to avoid non-steroidal anti-inflammatory drugs (NSAIDs) like aspirin and ibuprofen, as they can heighten the risk of bleeding complications [25]. Severe cases of dengue fever may necessitate hospitalization for intensive supportive care, intravenous fluid and electrolyte replacement, blood pressure monitoring, and transfusions if warranted [25,28]. Preventive measures against dengue fever include protecting oneself from mosquito bites and, in endemic areas, vaccination with the CYD-TDV dengue vaccine [25]. These strategies aim to mitigate the risk of dengue virus transmission and reduce the burden of dengue fever on affected populations.

Association between dengue infection and HLH

Case Studies and Clinical Reports

Several case reports have documented patients developing hemophagocytic lymphohistiocytosis (HLH) as a rare complication of dengue infection [29-32]. HLH is characterized by persistent fever, pancytopenia, hepatosplenomegaly, and elevated serum ferritin levels. The association between dengue and HLH is well-established, with numerous similarities between the two conditions, such as a notable increase in cytokine levels and thrombocytopenia [29]. Dengue-induced HLH can significantly impact the progression of dengue infection and result in serious adverse outcomes if not promptly recognized and treated [30]. Case reports have depicted instances of both primary HLH triggered by dengue infection and secondary HLH developing as a complication of severe dengue [31]. These reported cases underscore the critical importance of early recognition and appropriate therapy, including the administration of dexamethasone and etoposide, to enhance patient outcomes in dengue-associated HLH [32].

Mechanisms Underlying HLH in Dengue Infection

The dysfunction of cytotoxic T cells and natural killer (NK) cells is a central feature of hemophagocytic lymphohistiocytosis (HLH). This dysfunction precipitates the uncontrolled proliferation and activation of lymphocytes and histiocytes, which correlates with the phagocytosis of hematopoietic cells [33]. Viral infections, including dengue, commonly serve as triggers for the development of secondary or acquired HLH. However, the precise mechanisms through which viruses like dengue contribute to the pathogenesis of HLH remain unconfirmed [34]. The prevailing theory posits that inappropriately proliferating and activated T cells play a pivotal role in the genesis of HLH in dengue infection. This phenomenon results in excessive cytokine production and the hyperinflammatory state characteristic of HLH [32]. Additionally, genetic factors and co-infections, such as Plasmodium vivax malaria, may influence the development of HLH in dengue patients. However, the relative contributions are not definitively established [33].

Risk Factors and Predictors

Several factors have been identified as potential predictors for the development and prognosis of hemophagocytic lymphohistiocytosis (HLH) in the context of severe dengue infection. Firstly, prolonged fever duration has emerged as a significant factor, with studies indicating that a fever lasting over 12.5 days correlates with a heightened risk of HLH development [35]. Additionally, elevated triglyceride levels exceeding 3.02 mmol/L have been associated with an increased likelihood of HLH occurrence in severe dengue cases [35]. Moreover, advancing age has been recognized as a risk factor for poorer outcomes among adult patients with secondary HLH, including those with dengue-associated HLH [36]. Furthermore, coagulation abnormalities, such as a low platelet count (<40 x 10^9/L), have been linked to heightened mortality rates in adult patients with secondary HLH [36,37]. Elevated levels of liver enzymes (AST), lactate dehydrogenase (LDH), and creatinine have also been identified as indicators of organ dysfunction, with their presence associated with a worse prognosis in adult secondary HLH cases [36]. Additionally, hyperferritinemia, characterized by persistently elevated serum ferritin levels, a hallmark of HLH, has been identified as a poor prognostic factor in adult secondary HLH [37]. These factors collectively underscore the complexity and severity of HLH in the context of dengue infection, emphasizing the importance of vigilant monitoring and early intervention in affected individuals.

Challenges in Diagnosis and Treatment

Diagnosing hemophagocytic lymphohistiocytosis (HLH) poses a considerable challenge, particularly in critically ill patients, as its clinical presentation is diverse and may resemble other inflammatory disorders [38,39]. The absence of specific diagnostic tests necessitates that clinicians rely on a combination of clinical and laboratory findings, often leading to diagnostic delays [38,39]. Although hemophagocytosis in the bone marrow or other tissues may suggest HLH, its presence is not obligatory for diagnosis and can also be observed in other conditions. Differentiating between "true" HLH and "HLH disease mimics" is crucial for enhancing recognition and management strategies [38,39]. Timely initiation of appropriate immunochemotherapy is paramount for patient survival; however, the rarity of HLH and its variable presentation may impede timely diagnosis and treatment [40]. The dynamic clinical course, coupled with the high risk of treatment-related morbidity and the potential for disease recurrence, further complicates HLH management [40]. In the specific context of dengue-associated HLH, mortality rates can be alarmingly high, reaching up to 78% in certain studies, underscoring the urgency of early recognition and aggressive treatment measures.

Clinical management of HLH-dengue syndrome

Treatment Guidelines for HLH

Early recognition and diagnosis are pivotal in managing hemophagocytic lymphohistiocytosis (HLH). The guidelines established by the Histiocyte Society outline specific criteria for identifying and diagnosing HLH, encompassing manifestations such as fever, splenomegaly, cytopenias, hypertriglyceridemia, hemophagocytosis, hyperferritinemia, impaired NK cell function, and elevated soluble CD25 (sCD25) [41,42]. Immunosuppressive therapy forms the cornerstone of HLH treatment, with high-dose corticosteroids such as dexamethasone or methylprednisolone being commonly employed to suppress the hyperinflammatory response [41,43]. In severe cases or instances of poor response to steroids, additional therapies such as etoposide may be recommended, particularly in adult HLH patients without concurrent macrophage activation syndrome (MAS) [41]. Supportive care constitutes an integral component of HLH treatment, encompassing fluid resuscitation, blood product transfusions, and the management of associated complications [42,43]. Continuous monitoring of patients is imperative, with treatment regimens potentially requiring adjustments based on clinical response and laboratory parameters [43]. Given the risk of acquired cellular immunodeficiency associated with HLH-directed therapy, patients may necessitate broad antimicrobial prophylaxis against infections like Pneumocystis jirovecii and fungi. Additionally, considerations for antiviral prophylaxis and hospitalization in units equipped with high-efficiency particulate air-filtered air may be warranted [44]. These prophylactic measures aim to mitigate the risk of opportunistic infections and optimize patient outcomes during HLH treatment.

Specific Considerations in the Context of Dengue Infection

Early recognition of hemophagocytic lymphohistiocytosis (HLH) is paramount in dengue patients, particularly when persistent fever, pancytopenia, hepatosplenomegaly, and markedly elevated serum ferritin levels are present [34]. Clinicians should maintain a high index of suspicion for HLH in these cases to facilitate timely diagnosis and intervention. The cornerstone of treatment for dengue-associated HLH lies in the prompt initiation of immunosuppressive therapy. Early administration of high-dose corticosteroids, such as intravenous dexamethasone or methylprednisolone, is crucial for suppressing the hyperinflammatory state characteristic of HLH [25,34]. Supportive management forms an integral component of the treatment approach for dengue-HLH. This encompasses fluid resuscitation, blood product transfusions, and meticulously managing associated complications to optimize patient outcomes [34]. In severe cases of dengue-HLH accompanied by organ dysfunction or an inadequate response to steroids, consideration of additional immunomodulatory agents such as intravenous immunoglobulin (IVIG) or chemotherapeutic agents like etoposide may be warranted [25]. Continuous monitoring of patients is essential throughout the treatment process, with regular assessment of clinical response and laboratory parameters guiding adjustments to the therapy regimen as necessary [25]. Recognizing the importance of early recognition and prompt treatment, clinicians must remain vigilant in identifying dengue-HLH as a potentially life-threatening condition. Initiating appropriate therapy without delay can significantly improve patient outcomes and potentially avert fatal consequences [34].

Supportive Care and Complication Management

Fluid resuscitation is a cornerstone in managing patients with hemophagocytic lymphohistiocytosis (HLH), crucial for maintaining hemodynamic stability and averting complications such as organ dysfunction [45]. Adequate fluid management is pivotal in optimizing patient outcomes by ensuring adequate tissue perfusion and preventing hypovolemia-related complications. In addition to fluid resuscitation, patients with HLH often necessitate transfusions of blood products to address the associated pancytopenia and coagulopathy. This typically includes packed red blood cells, platelets, and fresh frozen plasma to address the multifaceted hematological challenges [45]. Such transfusions are instrumental in mitigating the risk of bleeding and optimizing hemostasis. Close monitoring and management of organ dysfunction are paramount to the comprehensive care of HLH patients. This entails vigilant surveillance and timely intervention for conditions such as acute kidney injury, liver dysfunction, and respiratory failure, which may arise as sequelae of HLH pathology [45]. Effective management of organ dysfunction is essential for mitigating the morbidity and mortality associated with HLH. Given the heightened susceptibility to infections in HLH patients, empiric broad-spectrum antimicrobial therapy may be warranted to manage underlying or secondary infections [45]. Prompt initiation of antimicrobial therapy is vital for preventing infectious complications and optimizing patient outcomes in HLH. In severe respiratory distress or failure cases, supportive ventilation measures such as mechanical ventilation may be indispensable for maintaining adequate oxygenation and ventilation [45]. These interventions are essential for supporting respiratory function and preventing respiratory compromise in critically ill HLH patients. Continuous patient monitoring is imperative throughout treatment, with regular assessment of clinical response and laboratory parameters guiding adjustments to the supportive care regimen as needed [45]. This iterative approach ensures therapeutic interventions are tailored to the patient's needs, optimizing treatment efficacy and enhancing patient outcomes.

Future perspectives and research directions

Emerging Insights into HLH-Dengue Association

Hemophagocytic lymphohistiocytosis (HLH) represents a rare yet potentially life-threatening complication of dengue infection, characterized by persistent fever, pancytopenia, hepatosplenomegaly, and elevated serum ferritin levels [46]. Dysfunction of cytotoxic T cells and natural killer cells underlies the pathogenesis of HLH, leading to uncontrolled lymphocyte and histiocyte activity. Infections such as dengue and Plasmodium vivax are infrequent triggers of HLH [33]. The clinical presentation of HLH encompasses prolonged fever, a sepsis-like syndrome, cytopenias, elevated serum aminotransferase levels, hypofibrinogenemia, hypertriglyceridemia, hyperferritinemia, and increased lactic dehydrogenase levels. While dengue and malaria are recognized as significant but uncommon causes of HLH [33], diagnosing HLH in dengue patients poses challenges due to overlapping clinical features. Hence, maintaining a high index of clinical suspicion is imperative for facilitating timely diagnosis and treatment [46]. Despite its life-threatening potential, HLH associated with dengue infection is amenable to treatment. Prompt initiation of appropriate therapy, including dexamethasone and etoposide, improves patient outcomes [47]. However, patients with severe dengue may develop secondary HLH, carrying a notable risk of mortality. Clinical indicators such as elevated levels of aspartate aminotransferase, alanine aminotransferase, lactate dehydrogenase, and creatinine are associated with increased fatality in these cases [47]. Thus, early recognition and intervention are critical in mitigating the mortality risk associated with dengue-related HLH.

Potential Therapeutic Strategies

Hemophagocytic lymphohistiocytosis (HLH) represents a hyperinflammatory condition that can pose life-threatening complications. HLH-directed therapy, incorporating medications like dexamethasone and etoposide, has demonstrated promising efficacy in reducing mortality rates among patients with severe dengue infection complicated by HLH. Initiation of this treatment regimen promptly is paramount for enhancing patient outcomes [34,47]. In instances where patients with dengue-associated HLH manifest organ dysfunction, immunotherapy may be warranted. This therapeutic approach may entail the administration of systemic corticosteroids, intravenous immunoglobulin (IVIG), or a combination of both to modulate the immune response and mitigate the hyperinflammatory state associated with HLH [34]. Supportive care constitutes a critical component of the treatment strategy for dengue-associated HLH. This encompasses monitoring and managing complications, providing blood products as necessary, and ensuring overall patient stability while undergoing specific HLH-directed therapies [47]. Early recognition and diagnosis of HLH in dengue patients are imperative for timely intervention. Clinicians should maintain a heightened level of suspicion for HLH in dengue patients, particularly when confronted with persistent fever, cytopenias, or markedly elevated serum ferritin levels. Timely diagnosis facilitates the prompt initiation of appropriate treatment modalities [34,47]. Regular clinical monitoring, including assessments of liver function, renal function, hematological parameters, and serum ferritin levels, is integral to the management of dengue-associated HLH. Close monitoring aids in evaluating the response to treatment and detecting any potential complications that may arise during the therapeutic course [34,47].

Areas for Further Investigation

Further research is imperative to develop more specific and sensitive diagnostic tools and criteria for identifying hemophagocytic lymphohistiocytosis (HLH) in patients with dengue infection. Enhancing the accuracy and efficiency of diagnosis can facilitate earlier recognition and appropriate management of this complication [30,46]. Understanding the underlying mechanisms by which dengue infection triggers the development of HLH is critical. Investigating the pathogenesis and identifying specific risk factors associated with dengue-induced HLH can provide valuable insights into preventive strategies and targeted treatments [30,33]. Efforts to optimize treatment strategies for dengue-associated HLH through research are paramount. Studying the efficacy and safety of various therapeutic approaches, including immunosuppressive agents like dexamethasone and etoposide, can significantly enhance patient outcomes and mitigate mortality rates [30,33]. Exploring prognostic factors influencing the outcomes of patients with dengue-associated HLH is essential. Identifying specific clinical, laboratory, and genetic markers predicting disease severity and treatment response can inform personalized management strategies and enhance patient care [33,47]. Conducting comprehensive epidemiological studies to ascertain the incidence, prevalence, and clinical characteristics of dengue-associated HLH is crucial. Accumulating more data on the epidemiology of this rare complication can deepen our understanding of its impact and guide public health interventions effectively [33,47]. Investigating the role of genetic factors in predisposing individuals to dengue-induced HLH represents an area deserving further exploration. Understanding the genetic susceptibility to this complication can aid in identifying at-risk populations and tailoring treatment approaches accordingly [33,47].

## Conclusions

In conclusion, this comprehensive review has shed light on the intricate interplay between hemophagocytic lymphohistiocytosis (HLH) and dengue infection. We have delved into the underlying pathophysiological mechanisms driving HLH in the context of dengue, discussed the diverse clinical manifestations and diagnostic challenges encountered, and explored various therapeutic strategies. Throughout this exploration, it has become evident that early recognition and management of HLH-dengue syndrome are paramount for improving patient outcomes. Healthcare providers must maintain a heightened suspicion of HLH in patients with dengue, especially those presenting with unexplained cytopenias, hyperferritinemia, and multiorgan dysfunction. Timely initiation of appropriate diagnostic tests, including bone marrow examination and genetic studies, is crucial for accurate diagnosis and prompt treatment initiation. Moreover, interdisciplinary collaboration among infectious disease specialists, hematologists, and critical care teams is essential to managing HLH-dengue syndrome comprehensively. However, despite advances in our understanding, the complexity of this syndrome remains a formidable challenge, necessitating further research to elucidate underlying mechanisms, refine diagnostic criteria, and explore novel therapeutic interventions.
